# The Relationship between Stress Perception and Problematic Social Network Use among Chinese College Students: The Mediating Role of the Fear of Missing Out

**DOI:** 10.3390/bs13060497

**Published:** 2023-06-13

**Authors:** Wangqian Fu, Rui Li, Yuqian Liang

**Affiliations:** 1 Faculty of Education, Beijing Normal University, Beijing 100875, China; 2 School of Education, Beijing Sport University, Beijing 100091, China; 3 School of Social Development and Public Policy, Beijing Normal University, Beijing 100875, China

**Keywords:** stress perception, problematic social network use, fear of missing out, Chinese college students

## Abstract

Based on the interaction of person-affect-cognition-execution model, this study examined the association between stress perception and problematic social network use among Chinese college students and explored the mediating effect of the fear of missing out (FoMO) on the relationship between stress perception and problematic social network use. A questionnaire survey was conducted among 554 students from nine universities in China. We found that stress perception was significantly positively correlated with problematic social network use and FoMO (r = 0.38, 0.46; *p* < 0.001), and FoMO was significantly positively correlated with problematic social network use (r = 0.45, *p* < 0.001). FoMO mediated the relationship between stress perception and problematic social network use. Conclusion: Stress perception has a negative impact on college students’ problematic social network use via the mediating effect of FoMO. Practical implications that reveal the college students’ problematic social network use were discussed as well.

## 1. Introduction

With the rapid development of social networks and information technology and the convenience of interpersonal communication, people’s communication methods are increasingly transferred to online platforms, and more people hope to establish relationships with others using social networks [1,2]. Social network use has become an important part of individuals’ daily lives, especially for teenagers and young adults [3,4,5]. By using social network platforms, young people can display their own status and experiences, find and share information and materials, enhance relationships with friends, strengthen their self-identity, construct a social identity, obtain a sense of belonging, and meet their psychological needs [6,7,8,9]. In addition to its positive effects, social networking can also have negative psychological and behavioral effects on children, adolescents, and young adults due to the excessive use of social networking [10,11,12,13]. Young people who use social networks too much are at risk of developing mental health problems, including addiction, loneliness, and depression [14,15,16]; have worse sleep quality [17,18,19,20,21]; and are at risk of cyberbullying, harassment, stalking, and victimization [22,23]. In addition, people who are heavily involved in social media have less time to pursue other healthy activities and hobbies and to spend with their families [24]. It has been noted that the symptoms of problematic social network use coincide with those of behavioral and substance addiction, i.e., withdrawal, conflict, tolerance, relapse, salience, and mood changes [25].

According to the latest official report released by the China Internet Network Information Center (CNNIC), there are more than 978 million Internet users in China, among which college students are the main group [26]. In China, college students mainly use WeChat, Weibo, and other social media, and 90% of them are active users of WeChat [27]. In this regard, problematic social network use by Chinese college students has attracted the attention of many researchers [28,29,30]. The problematic social network use of college students is a phenomenon of compulsive behavior characterized by psychological distress and physiological discomfort experienced by individuals who spend more time than normal using social networks at a high intensity; this high-intensity use is difficult to limit and irresistible to them [31,32]. Problematic social network use may have a disastrous impact on the mental and physical health of college students and needs to be taken seriously [33]. Thus, it is worthwhile to explore the formation mechanism of the problematic social network use of college students.

### 1.1. College Students’ Stress Perceptions and Problematic Social Network Use

Stress perception occurs when people perceive a situation to be threatening or otherwise demanding and lack sufficient resources to deal with the situation [34]. College students often experience a variety of pressures, such as academic and school, peer, and family pressures [35,36], which may lead to a relatively high level of stress perception [37]. Stress perception is an important factor in the occurrence and relapse of many addictive disorders [38,39]. According to general strain theory [40], perceived pressure produces negative emotions, and people will choose to escape stressful situations: The higher the perceived pressure, the more inclined they are to adopt negative coping styles. Some studies have found that perceived stress can cause individuals to be prone to out-of-control behaviors, which may lead to problematic Internet use [41]. Evidence shows that there is a positive correlation between stress perception and problematic Internet use [42,43]. On the one hand, college students may use social networks excessively to relieve pressure and eliminate negative emotions [44,45,46]. On the other hand, some students may deal with problems passively and spend substantial time on the Internet to avoid solving problems [47]. In conclusion, stress perception can positively predict problematic social network use [48]. Therefore, Hypothesis 1 is proposed in this study: stress perception can negatively predict problematic social network use among college students.

### 1.2. Stress Perception, Fear of Missing Out, and Problematic Social Network Use in College Students

Fear of missing out (FoMO), missing fear, or fear of missing is the dispersive emotional experience of anxiety, fear, loss, worry, depression, and other negative feelings generated by individuals that are concerned with missing important information, novel events, or the beneficial experiences of others [49,50], and it can be regarded as a subclass of anxiety [51]. According to the interaction of person-affect-cognition-execution model (I-PACE model) [52], user traits (stress perception) can be used as the inducing variable, subjective perception (miss anxiety) can be used as the mediating variable, and implementation (problematic social network use) can be used as the outcome. Brand et al. (2016) reckoned that the I-PACE theoretical model architecture can effectively analyze the dynamic generation mechanism process of “psychology → behavior” of mobile social media FoMO users [52]. To be specific, the influencing factors of the fear of missing out include personality traits, psychological needs, situational factors, and interpersonal relationships [53,54,55], which can be understood as the phenomenon caused by the lack of satisfaction of individual psychological needs due to the information pressure generated by others [49]. FoMO may ultimately increase negative emotions and symptoms and affect personal well-being [56,57]. Moreover, some evidence shows that stress perception positively predicts FoMO [58,59,60].

Previous studies have shown a significant correlation between FoMO and problematic social network use [61,62,63]. The higher an individual’s FoMO, the more serious their problematic social network use [13,55,64,65,66,67,68,69]. Furthermore, FoMO can not only effectively predict the frequency and intensity of people’s use of social networks [49,54,62,70] but also predict metacognition related to social network use, which in turn predicts problematic social network use [71,72,73]. These findings suggest that stress perception, FoMO, and problematic social network use are closely related among college students. It is worth noting that many studies have confirmed the correlation between FoMO and a variety of psychological factors, including negative emotions [74], anxiety [66], and depression [65]. Meanwhile, FoMO has also been found to mediate the relationship between a series of psychological factors and social network use [75,76,77,78]. Therefore, Hypothesis 2 is proposed in this study: FoMO partially mediates the relationship between stress perception and problematic social network use among college students.

### 1.3. The Current Study

Problematic social network use is an important risk factor leading to social anxiety and depression symptoms in individuals, and it has a significant impact on individual mental health [79]. The study of college students’ problematic social network use needs to be strengthened and expanded upon since it is an inevitable topic in the growth of college students. Although the relationship between college students’ stress perceptions and problematic social network use has been established, the understanding of the mechanism underlying this relationship is not yet sufficient. In particular, the association of FoMO in the relationship between stress perception and problematic social network use has rarely been examined in the studies of Chinese college students [51]. In accordance with the I-PACE theoretical paradigm, execution (problematic social network use) was the outcome variable, with user attributes (stress perception) serving as the mediating variable. As a result, a mediating model was created that took into account FoMO in the link between stress perception and problematic social network use in order to investigate the precise psychological mechanisms involved in college students’ perceptions of stress and the use of problematic social networks. The study’s findings should help reduce college students’ stress, preserve their physical and mental health, and ensure that social media is used responsibly.

## 2. Method

### 2.1. Participants and Procedure

The subjects of this study are college students from 9 universities in Beijing, Henan Province, and Yunnan Province. We contacted instructors at those universities and asked for help in sending the questionnaire link to their students. A total of 650 college students were selected to participate in the questionnaire survey using the convenience sampling method, and 554 valid questionnaires were collected (85.23%). Among them, 449 (80.90%) were female; there were 238 freshmen, 165 sophomores, 48 juniors, and 103 seniors. The subjects ranged in age from 16 to 25 years (*M* = 19.55 years; *SD* = 1.43 years).

### 2.2. Measures

#### 2.2.1. Stress Perception

The English version of the stress perception scale compiled by Cohen et al. [34] was adopted, and the Chinese version measured by Chu et al. [21] in Chinese college students was also used. There are 10 items in the scale, and each item is rated from 0 (never) to 4 (always). The Cronbach’s α coefficient of the questionnaire in this study was 0.76.

#### 2.2.2. Fear of Missing Out

We used the Chinese version of the trait-state fear of missing out scale (T-SFoMOS) compiled by Beaton et al. [80] and translated by Li et al. [81]. It has also been shown to be suitable for Chinese college students (Li et al., 2020). There were 12 items in the scale in two dimensions: trait-FoMOS (5 items) and state-FoMOS (7 items). Each item was rated from 1 (completely disagree) to 5 (completely agree). In this study, the Cronbach’s α coefficient of the questionnaire was 0.94.

#### 2.2.3. Problematic Social Network Use

The Chinese short form of the social media disorder scale (SMDS) compiled by van den Eijnden et al. [82] and translated by Zhang et al. [83] was applied in the study. There are 9 items in the scale, representing 9 dimensions. The items are answered as “yes/no” (0 = “no”, 1 = “yes”), and the score ranged from 0 to 9. According to the cutoff point for IGDs in the DSM-5, a score of 5 or above is considered problematic social network use [84]. The Cronbach’s α coefficient of the questionnaire in this study was 0.90.

### 2.3. Data Analysis

We examined the data distribution first, and then AMOS 26.0 and SPSS 25.0 were used to conduct descriptive statistical analysis, common method bias tests, confirmatory factor analysis, reliability analysis, and correlation analysis of the collected data. On this basis, Model 4 in the SPSS PROCESS macro was further used to test the mediation effect model by estimating the 95% confidence interval of the mediation effect via 5000 sample samples.

## 3. Results

### 3.1. Common Method Bias

For possible common method deviation in data collection using the self-report method, based on program control (such as anonymous method filling, reverse scoring of some items, etc.) [85], the Harman single-factor test was used for the common method deviation test. A total of five factors with characteristic roots greater than 1 were extracted from the results of unrotated exploratory factor analysis, and the maximum factor variance interpretation rate was 35.48% (less than 40%). Meanwhile, confirmatory factor analysis was used to conduct a common method deviation test for all self-rated items [86], and the results showed that the model fit was poor: χ2/df = 7.47; CFI = 0.76; IFI = 0.76; TLI = 0.74; RMSEA = 0.11; RMR = 0.11. Therefore, there is no serious common method bias in the measurement [87].

### 3.2. Descriptive Analysis and Variance Testing among Stress Perception, FoMO, and Problematic Social Network Use

According to the descriptive statistics and correlation analysis of stress perception, FoMO, and problematic social network use, the results showed that the variables could be accepted as normally distributed. The mean value of pressure perception was 1.77 (kurtosis = 0.77; skewness = −0.10). The mean value of FoMO was 2.73 (kurtosis = 0.92; skewness = −0.07), and the mean value of problematic network use was 0.25 (kurtosis = 0.28; skewness = 1.12) (see Table 1).

### 3.3. Variance Testing for Gender among Stress Perception, FoMO, and Problematic Social Network Use

A *t* test was applied to examine the differences in stress perception, FoMO, and problematic social network use among students of different genders (See Table 2). We found that the average score for the pressure perception of male students was higher than that of female students (*t* = 2.02, *p* < 0.05), and the other variables had no significant difference by gender.

### 3.4. Variance Testing with Respect to Grades among Stress Perception, FoMO, and Problematic Social Network Use

An F test was used to examine the differences in stress perception, FoMO, and problematic social network use of students across grades (Table 3). The problematic social network use and FoMO of college students were not significantly different by grade level, while the stress perception of college students was significantly different by grade level. Therefore, a comparative analysis was conducted after the event. As observed in the results of the postcomparative analysis (least significant difference, LSD), the pressure perception scores of senior students were higher than those of freshmen and sophomores. The stress perception scores of juniors were higher than those of freshmen.

### 3.5. Correlations between Stress Perception, FoMO, and Problematic Social Network Use

The correlation analysis showed that there was a significant positive correlation between stress perception and problematic social network use (*r* = 0.38, *p* < 0.001), and there was a significant positive correlation between perceived stress and FoMO (*r* = 0.46, *p* < 0.001). There was a significant positive correlation between FoMO and problematic social network use (*r* = 0.45, *p* < 0.001) (see Table 4). This result indicated that the problematic social network use of college students increased with an increase in perceived stress and FoMO, and FoMO also increased with an increase in perceived stress level.

### 3.6. The Mediating Role of FoMO

**Figure 1:**
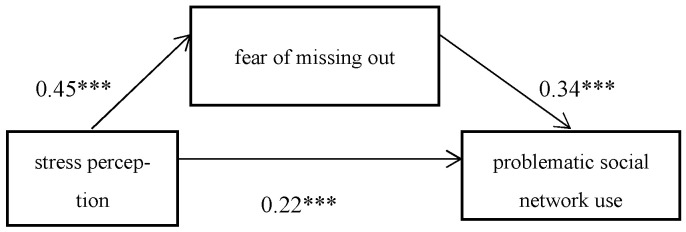
The mediating effect of FoMO. Note: *** *p* < 0.001.

Using the SPSS PROCESS Model 4 macro program (Model 4 is a simple mediation model) compiled by Hayes (2012), the percentile bootstrap method with deviation corrections was used to test the mediating effect of the FoMO on the relationship between perceived stress and problematic social network use, and gender and grade were used as control variables. The results showed that (see Table 5) perceived stress was significantly associated with the problematic use of social networks (*Β* = 0.38, *t* = 9.38, *p* < 0.001), and after taking FoMO as the intermediary variable, the perceived stress of problematic social networks using a direct prediction effect was still significant (*Β* = 0.22; *t* = 5.21; *p* < 0.001). The relationship between perceived stress and FoMO was significant (*Β* = 0.45; *t* = 11.71; *p* < 0.001), and the association between FoMO and problematic social network use was significant as well (*Β* = 0.34; *t* = 8.23; *p* < 0.001). In addition, both the upper and lower limits of the bootstrap 95% confidence interval for the direct effect of stress perception on problematic network use and the mediating effect of FoMO did not include 0 (see Table 6), indicating that stress perception can not only be associated with problematic social network use but also included problematic network use via the mediating effect of FoMO. The direct effect (0.12) and the intermediate effect (0.09) accounted for 57.14% and 42.86% of the total effect (0.26), respectively (see Figure 1).

## 4. Discussion

This study explored the correlations among stress perception, FoMO, and problematic social network use among Chinese college students. The results showed that stress perception was positively correlated with FoMO and problematic social network use. Additionally, FoMO played a mediating role in the relationship between stress perception and problematic social network use among Chinese college students. The findings provide potential intervention strategies for improving problematic social network use among Chinese college students.

### 4.1. Relationships between Stress Perception, Problematic Social Network Use, and FoMO in Chinese College Students

We found there was a positive correlation between students’ stress perception and problematic social network use. It is consistent with previous findings on the relationship between college students’ stress perception and problematic social network use [29,88,89]. The more pressure college students experience, the more likely they are to use problematic social networks [90]. College student experience pressure caused by the external environment, such as family economic status, interpersonal relationships, and career choice, which may lead to problematic social network use among college students [91]. This can be explained by the compensatory Internet use model proposed by Kardefelt-Winther [31]. Stressful situations tend to lead to self-depletion and psychological needs [92], while satisfying psychological needs is the driving force for individual actions. If it is difficult to compensate for their self-needs, they shift their goals to the network world [93]. Alternatively, the intemperance of self-compensation leads to symptoms such as Internet addiction [94]. In other words, college students under the stress perception condition are more likely to exhibit out-of-control behaviors, so problematic social network use is more likely to occur.

Second, there is a significant positive correlation between college students’ stress perception and FoMO, which is consistent with previous findings [95,96]. Fundamentally, FoMO is an emotional problem, and the perceived pressure affects its level [59]. The degree of anxiety and depression is positively correlated with FoMO [71,78,97], and stress is the most important variable for predicting the level of anxiety [98,99]. The greater the perceived pressure, the more likely the individual is to experience a sense of tension and loss of control, and the higher the anxiety level [59]. In addition, stressful events of sensory and psychological stimulation in social networking sites can easily lead to stressful states in users’ psychological perception, thus affecting FoMO [100].

In addition, we found that FoMO was positively associated with problematic social network use, which has been confirmed by previous findings [101,102]. Addiction can be regarded as an impulse control disorder [103,104]. According to the limited self-control model, individuals’ self-control resources are limited in the short term. After a period of activities requiring self-control resources, resources are depleted, and the ego transitions into a state of weak control, which leads to ego depletion [24,97]. After ego depletion occurs, a lack of sufficient self-control resources induces addiction [105], while FoMO may cause ego depletion [28]. Individuals with high levels of miss anxiety are more likely to overuse social media [106,107], deepening their use of problematic social networks [108]. Individuals with a high level of FoMO tend to frequently browse instant information published on social platforms, so they are exposed to many situations or activities that they did not participate in, which further strengthens the belief of the individual with respect to missing information and further aggravates their level of anxiety [65,109,110,111].

### 4.2. The Mediating Role of FoMO

This study showed that FoMO mediated the relationship between stress perception and problematic social network use among college students. According to the I-PACE model, specific Internet-use disorders are considered to be a consequence of interactions between predisposing factors, such as stressful situation, and mediators, such as affective and cognitive responses to situational triggers in combination with reduced executive functioning (e.g., FoMO) [52]. Perceived stress may result in personal conflicts or abnormal moods (e.g., FoMO). The FoMO makes individuals eager to know the dynamics of others all the time and stay informed about what others are doing, which may prompt individuals to formulate specific behaviors [49]. The FoMO responses to situational factors may influence whether individuals decide to use the Internet potentially to cope with the associated cognitions and effects [112]. FoMO as a cognitive factor is related to features of Internet-use disorders in combination with Internet-related expectancies or even illusions (i.e., false beliefs about the effects of using certain applications/sites [113] as well as implicit associations). These psychological defects may induce and promote problematic social network use [76,111]. Franchina et al. found that individuals experiencing a high level of FoMO may encounter problematic social network use [114] and suffer as a result. Fuster et al. claimed that FoMO could drive social media use [115].

## 5. Practical Implications

The findings of this study have implications for college students with problematic social network use.

First, perceived stress is an important predictor of problematic social network use. Perceived stress can be reduced by encouraging college students to participate in social activities and sports, providing mental health services [116], enhancing mental resilience [29], etc. Colleges and universities should pay attention to the influence of various aspects of stress on the mental health and social adaptation of college students. In particular, colleges and universities should pay close attention to the psychological development and behavior of college students and implement timely preventive and intervention measures.

Second, FoMO plays an important role in the relationship between college students’ stress perception and problematic social network use. Attention should be given to reducing the FoMO of college students, implementing relevant intervention measures, and providing professional education training and social support for college students. Meanwhile, the interactions between core personal, cognitive, and affective features should be considered more systematically in intervention strategies.

## 6. Limitations and Future Direction

There are still some limitations in the current study. First, the questionnaires used in this study were self-reported questionnaires, which may have potential bias. Further research may consider using multiple methods or gathering multiple sources to reduce these biases. Second, the participants were from normal universities, so the gender ratio was unbalanced in the study. Future studies may consider expanding the sample range to balance the gender ratio. Third, the association of perceived stress on problematic social network use may vary across cultures. Future studies should include participants from various cultures to explore potential cultural differences.

## 7. Conclusions

In conclusion, this study confirms the seriousness of problematic social network use among Chinese college students. In addition, the correlation between stress perception, FoMO, and problematic social network use among college students was discussed, as well as the mediating effect of FoMO on the relationship between stress perception and problematic social network use among college students. The results showed that stress perception was positively correlated with FoMO and problematic social network use. Attention should be directed toward reducing the stress perception of college students, implementing relevant interventions, and providing professional educational training and social support to college students in order to help reduce problematic social network use.

## Figures and Tables

**Table 1:** Descriptive analysis of variables (*n* = 554).

Variables	*M*	*SD*	Q1	Q2	Q3	Kurtosis	Skewness
stress perception	1.77	0.50	1.40	1.80	2.00	0.77	−0.10
FoMO	2.73	0.73	2.25	3.00	3.00	0.92	−0.07
problematic social network use	0.25	0.31	0.00	0.11	0.44	0.28	1.12

**Table 2:** The variance testing for gender among the three variables.

Variables	Gender	*n*	*M*	*SD*	*t*	Cohen’s
stress perception	male	105	1.86	0.47	2.02 *	0.22
	female	449	1.75	0.51		
FoMO	male	105	2.78	0.81	0.81	0.09
	female	449	2.72	0.71		
problematic social network use	male	105	0.26	0.35	0.50	0.04
	female	449	0.25	0.31		

Note: * *p* < 0.05.

**Table 3:** Variance testing for grades among the three variables.

Variables	Grade	*n*	*M*	*SD*	*F*	LSD
stress perception	1	238	1.70	0.50	5.05 **	④ > ①, ④ > ②, ③ > ①
	2	165	1.74	0.54		
	3	48	1.89	0.49		
	4	103	1.90	0.42		
FoMO	1	238	2.67	0.73	2.02	
	2	165	2.72	0.74		
	3	48	2.71	0.75		
	4	103	2.88	0.70		
problematic social network use	1	238	0.25	0.33	2.32	
	2	165	0.21	0.27		
	3	48	0.27	0.32		
	4	103	0.31	0.33		

Note: ** *p* < 0.01; ① = grade 1; ② = grade 2; ③ = grade 3; ④ = grade 4.

**Table 4:** Correlation analysis results of variables (*n* = 554).

Variables	1	2	3
stress perception	1		
FoMO	0.46 ***	1	
problematic social network use	0.38 ***	0.45 ***	1

Note: *** *p* < 0.001.

**Table 5:** The mediation of FoMO (*n* = 554).

Independent Variable	Model 1: Problematic Social Network Use			Model 2: FoMO			Model 3: Problematic Social Network Use		
	*Β*	*SE*	*t*	*Β*	*SE*	*t*	*Β*	*SE*	*t*
gender	0.01	0.29	0.29	−0.00	0.87	−0.02	0.01	0.28	0.31
grade	−0.00	0.10	−0.07	0.03	0.31	0.67	−0.01	0.10	−0.30
stress perception	0.38	0.02	9.38 ***	0.45	0.07	11.71 ***	0.22	0.02	5.21 ***
FoMO							0.34	0.01	8.23 ***
*R* ^2^	0.14			0.21			0.24		
*F*	30.28			48.56			42.39		

Note: *** *p* < 0.001. All variables in the model are substituted into the regression equation by using standardized variables. All values are rounded to two decimal places.

**Table 6:** Total effect, direct effect, and the mediating effect (*n* = 554).

	Effect	Boot Standard Error	Boot CI Lower Limit	Boot CI Upper Limit	Relative Effect Size
total effect	0.21	0.02	0.17	0.26	
direct effect	0.12	0.02	0.08	0.17	57.14%
mediation effect	0.09	0.01	0.06	0.12	42.86%

Note: The boot standard error, lower boot CI limit, and upper boot CI limit refer to the standard error and lower and upper 95% confidence intervals of the indirect effect estimated by the bias-corrected percentile bootstrap method, respectively.

## Data Availability

The datasets generated and analyzed during the current study are available from the corresponding author upon reasonable request.
